# Progressive Destructive Hypothyroidism Associated with Sunitinib Therapy: A Three-Year Case Analysis

**DOI:** 10.3390/jcm15020788

**Published:** 2026-01-19

**Authors:** Marcin Nosal

**Affiliations:** Collegium Medicum, Jan Dlugosz University in Częstochowa, Waszyngtona 4/8 Street, 42-200 Częstochowa, Poland; marcinnosal12@gmail.com

**Keywords:** hypothyroidism, platelet-derived growth factor receptor, renal cell carcinoma, sunitinib, thyroid dysfunction, tyrosine kinase inhibitors, vascular endothelial growth factor receptor

## Abstract

Sunitinib, a tyrosine kinase inhibitor (TKI) targeting vascular endothelial growth factor receptors (VEGFRs) and platelet-derived growth factor receptors (PDGFRs), is widely used in renal cell carcinoma. A broad spectrum of thyroid dysfunctions has been observed during TKI therapy, yet their mechanisms and clinical progression remain only partially explained. A longitudinal case analysis of a woman with metastatic clear-cell renal cell carcinoma treated with cyclical sunitinib therapy (4 weeks on, 2 weeks off) was performed. Thyroid function tests, clinical symptoms, and ultrasound imaging findings were evaluated over time and compared with treatment exposure and dose adjustments. Baseline thyroid function was normal. During the third cycle, thyroid-stimulating hormone (TSH) increased markedly (33.44–41.26 mIU/L), with free thyroid hormones initially remaining within reference limits. TSH fluctuations corresponded to treatment intervals before stabilising into persistent hypothyroidism requiring levothyroxine replacement. Thyroid ultrasound revealed progressive parenchymal destruction and a reduction in gland volume from 18 mL to approximately 2 mL over three years. Endocrine management enabled maintenance of biochemical euthyroidism, and systemic oncological treatment continued without interruption. Sunitinib treatment may lead to progressive destructive hypothyroidism. Routine surveillance of thyroid function is essential, and timely levothyroxine therapy facilitates continued anticancer treatment and symptom control.

## 1. Introduction

Systemic cancer treatment increasingly relies on targeted therapies designed to interfere with specific molecular pathways driving tumour growth and progression. These strategies include monoclonal antibodies directed against receptors or their ligands on the tumour cell surface, as well as small-molecule agents that act intracellularly. The latter group primarily consists of kinase inhibitors, particularly tyrosine kinase inhibitors (TKIs), which disrupt phosphorylation-dependent signalling processes essential for cell proliferation, angiogenesis, and survival. TKIs have become established therapeutic options in multiple malignancies and are now commonly applied in routine clinical practice [1,2]. Sunitinib is a multitargeted TKI that inhibits several receptor systems, including vascular endothelial growth factor receptors (VEGFRs) and platelet-derived growth factor receptors (PDGFRs).

Through potent antiangiogenic activity, it reduces tumour vascularisation and progression, particularly in renal cell carcinoma, gastrointestinal stromal tumours, and pancreatic neuroendocrine tumours [3]. However, this mechanism may also negatively affect thyroid vascular supply, promoting destructive inflammation, tissue atrophy, and permanent hypothyroidism. Recognition of thyroid dysfunction associated with TKIs has increased over the past two decades. The first case of sunitinib-induced hypothyroidism was described in 2005, followed by numerous reports confirming biochemical and clinical thyroid abnormalities in patients receiving TKIs [4,5]. Thyroid dysfunction may present as transient thyrotoxicosis, subclinical hypothyroidism, or overt and persistent disease requiring hormone replacement therapy. The incidence is considered highest during sunitinib treatment. Although the underlying mechanisms are not fully understood, proposed pathways include impaired thyroid microcirculation due to VEGFR/PDGFR inhibition, altered deiodinase activity affecting peripheral thyroid hormone metabolism, reduced thyroperoxidase function, disrupted iodine uptake, and potential dysregulation of the hypothalamic–pituitary–thyroid axis [6,7]. Clinically, thyroid dysfunction may follow a biphasic pattern, beginning with TSH fluctuations that later stabilise into persistent hypothyroidism. Symptoms such as fatigue, cold intolerance, cognitive slowing, and weight gain may be non-specific and easily misinterpreted as cancer-related fatigue or treatment toxicity. Regular thyroid function monitoring is therefore recommended both before initiation of sunitinib treatment and throughout therapy [8]. Importantly, dysthyroidism rarely necessitates discontinuation of anticancer treatment. In most cases, appropriate levothyroxine replacement ensures restoration of euthyroidism and uninterrupted oncological management.

A better understanding of the pathophysiology and clinical behaviour of TKI-related thyroid dysfunction may improve diagnostic and therapeutic decision-making. As targeted therapies continue to expand, awareness of endocrine complications will become increasingly important in multidisciplinary oncology.

The following case illustrates the progressive course of thyroid dysfunction in a patient receiving sunitinib for metastatic renal cell carcinoma, demonstrating a transition from early biochemical abnormalities to permanent hypothyroidism and marked thyroid volume reduction.

## 2. Case Presentation

A 57-year-old woman with no prior endocrine disorders was referred for evaluation due to a marked elevation in serum thyroid-stimulating hormone (TSH) levels, while free thyroxine (FT4) and free triiodothyronine (FT3) concentrations remained within reference limits. The patient’s family history included a probable goitre in her paternal grandmother and unspecified thyroid disease in a sister, although further clinical details were unavailable. There was no known family history of autoimmune thyroid disease. Her medical history included hypertension, hyperlipidaemia, superficial vein thrombosis, degenerative joint disease, and prior carpal tunnel surgery.

One year before admission, ultrasound imaging revealed a right kidney tumour, and subsequent diagnostics confirmed clear-cell renal cell carcinoma. Four months later, the tumour was surgically removed. Histopathological assessment demonstrated stage T1b disease (tumour > 4 cm but ≤7 cm, confined to the kidney), with no evidence of lymphovascular invasion and a microscopically positive surgical margin (R1). Regional lymph nodes were not assessable (Nx). Within one month, bone metastases were detected.

Systemic treatment with the multitargeted tyrosine kinase inhibitor (TKI) sunitinib was initiated in 6-week cycles (4 weeks on, 2 weeks off). Following the first cycle, treatment was temporarily interrupted for orthopaedic surgery addressing a fracture-threatening metastatic lesion in the right femur, treated with hip arthroplasty. The patient also received stereotactic radiotherapy to a rib metastasis (40 Gy in 5 fractions). Baseline thyroid function was normal (TSH: 1.94 mIU/L).

**Figure 1:**
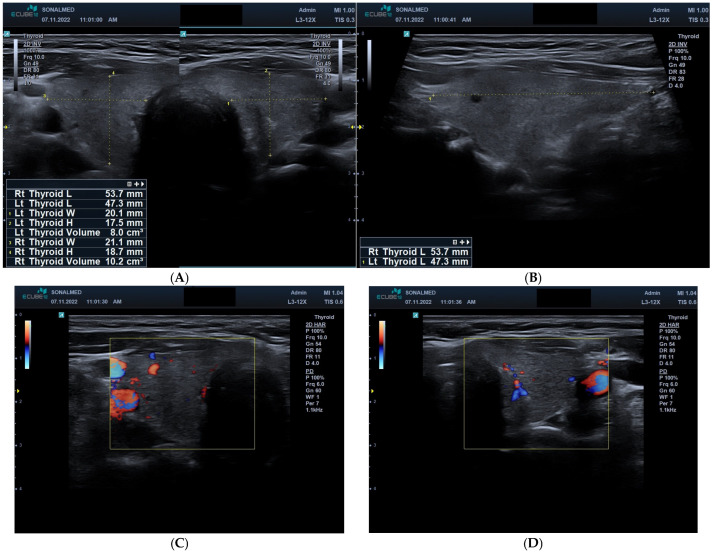
(**A**) Baseline transverse ultrasound image of the thyroid gland prior to sunitinib therapy. Grey-scale imaging shows both thyroid lobes with normal morphology and homogeneous parenchymal echotexture. Measurements indicate a left lobe volume of 8.0 cm^3^ and a right lobe volume of 10.2 cm^3^, consistent with values within the normal range. No focal lesions or structural abnormalities are visible. (**B**) Baseline transverse grey-scale ultrasound image of the thyroid gland. The image demonstrates both thyroid lobes in transverse view, showing normal parenchymal echogenicity and homogeneous internal structure. Measured thyroid lobe lengths (right: 53.7 mm; left: 47.3 mm) fall within the expected physiological range. No focal abnormalities or structural distortions are observed. (**C**) Baseline transverse colour Doppler ultrasound image of the thyroid gland prior to sunitinib therapy. The image shows a moderate number of intrathyroidal blood flow signals distributed focally within the thyroid parenchyma. Vascularisation is preserved, with no evidence of significant perfusion reduction or asymmetry between the lobes. (**D**) Baseline transverse colour Doppler ultrasound image of the thyroid gland prior to sunitinib therapy. Colour Doppler imaging demonstrates preserved intrathyroidal blood flow signals, visible as focal areas of vascularisation within the parenchyma. No significant perfusion defects or vascular asymmetry are observed, indicating normal baseline thyroid vascularity.

Baseline thyroid ultrasonography demonstrated normal gland morphology and volume, with left and right lobe volumes measuring 8.0 cm^3^ and 10.2 cm^3^, respectively, and lobe lengths (right: 53.7 mm; left: 47.3 mm) within the expected physiological range. The thyroid parenchyma showed homogeneous structure and normal echogenicity, with no focal lesions, distortions, or architectural abnormalities. Colour Doppler imaging confirmed preserved vascularisation, with focal intrathyroidal blood flow signals visible and no evidence of perfusion impairment or vascular asymmetry between the lobes. These baseline structural and vascular characteristics are demonstrated in Figure 1A–D, indicating a normal thyroid status prior to the initiation of sunitinib therapy.

## 3. Results

**Figure 2:**
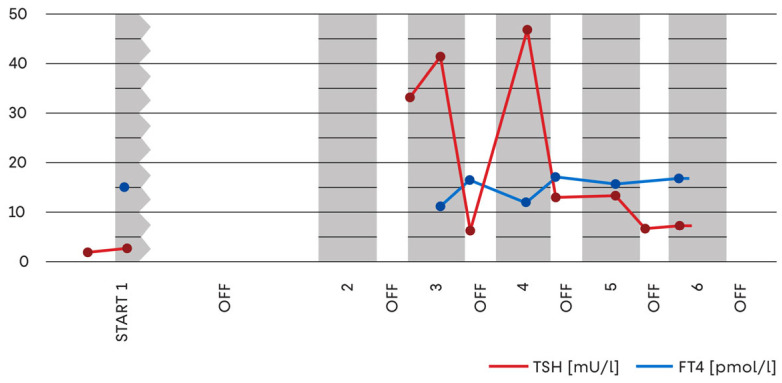
Serum TSH and FT4 concentrations during sunitinib therapy. The graph illustrates changes in thyroid-stimulating hormone (TSH) and free thyroxine (FT4) concentrations over sequential treatment cycles. Grey fields represent ON periods (drug administration), while white fields indicate OFF periods (drug holidays). The data show a rising trend in TSH values during active treatment cycles, accompanied by relatively stable FT4 concentrations, reflecting early biochemical thyroid dysfunction associated with sunitinib exposure.

During the third sunitinib treatment cycle, prior to radiotherapy, serum TSH levels increased markedly to 33.44–41.26 mIU/L, while FT4 and FT3 concentrations remained within reference limits. A clear upward trajectory of TSH values was observed (Figure 2).

The patient reported fatigue and general weakness but demonstrated no clinical features of overt hypothyroidism. Thyroid ultrasound at this stage revealed normal gland volume (18 mL), reduced vascularisation, and mildly heterogeneous parenchymal echotexture without focal abnormalities. Antithyroid antibodies were detectable at low titres (anti-thyroid peroxidase: 11.51 IU/mL; anti-thyroglobulin: 10.95 IU/mL). Sunitinib therapy was continued without modification.

Over the following cycles, TSH concentrations exhibited a cyclical pattern, characterised by spontaneous declines during sunitinib withdrawal intervals and renewed increases during ON-treatment periods, reflecting a reproducible association between drug exposure and thyroid dysfunction (Figure 2). As FT4 and FT3 concentrations initially remained stable, levothyroxine supplementation was deferred. However, persistent TSH elevation above 10 mIU/L at the end of treatment cycles prompted levothyroxine initiation at 25 μg/day during the fifth cycle.

**Figure 3:**
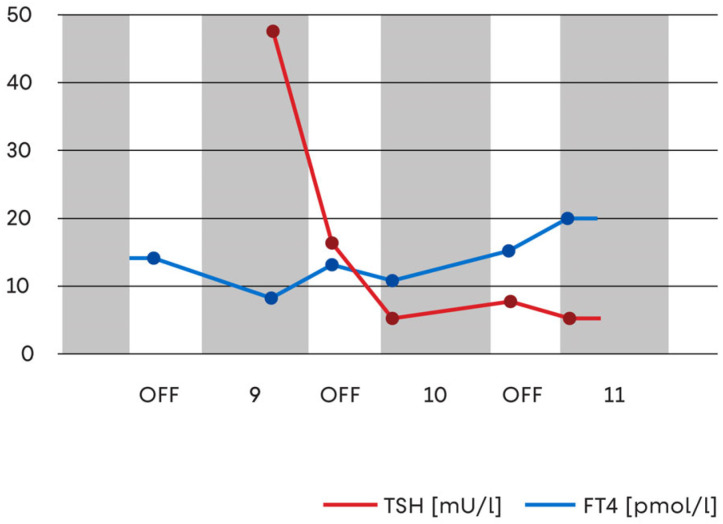
Serum TSH and FT4 concentrations during long-term sunitinib therapy. The graph shows thyroid-stimulating hormone (TSH) and free thyroxine (FT4) values across sequential treatment cycles. Grey fields correspond to ON periods (drug administration), while white fields represent OFF periods (drug holidays). A marked reduction in TSH levels and a gradual increase in FT4 concentrations are observed following the initiation of levothyroxine replacement therapy, indicating improved hormonal control while anticancer treatment continued.

A sustained biochemical progression toward overt hypothyroidism subsequently developed. FT4 values declined below reference limits and further levothyroxine dose adjustment was required, reaching 100 μg/day from cycle nine onward. This intervention resulted in hormonal stabilisation, evidenced by falling TSH and rising FT4 levels (Figure 3).

**Figure 4:**
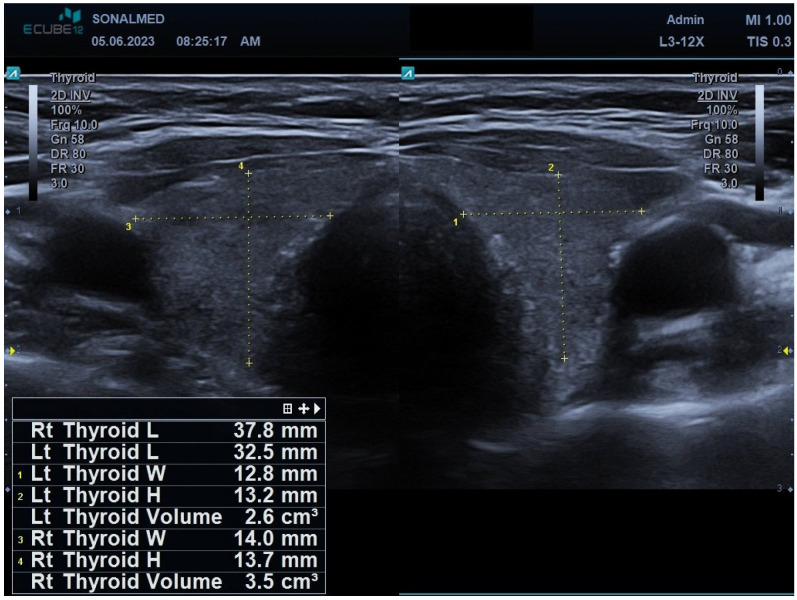
Transverse grey-scale ultrasound image of the thyroid gland after eight cycles of sunitinib therapy. The image demonstrates marked reduction in thyroid volume (left lobe: 2.6 cm^3^; right lobe: 3.5 cm^3^), accompanied by increased parenchymal heterogeneity and decreased echogenicity. These findings are consistent with progressive thyroid atrophy and structural deterioration associated with ongoing sunitinib treatment. Note: Yellow dotted lines—measurement markers delineating the thyroid boundaries for volumetric assessment of the gland.

Serial ultrasonography demonstrated progressive and severe thyroid involution. After eight cycles, total thyroid volume had decreased to approximately 6 mL, accompanied by increasing heterogeneity and reduced echogenicity, consistent with marked atrophic change (Figure 4).

**Figure 5:**
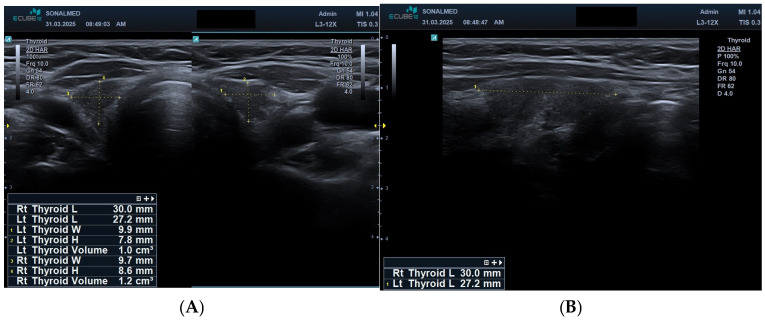
(**A**) Transverse grey-scale ultrasound image of the thyroid gland after three years of sunitinib therapy. The image shows a markedly reduced thyroid volume (left lobe: 1.0 cm^3^; right lobe: 1.2 cm^3^) with substantial parenchymal loss, decreased echogenicity, and pronounced structural heterogeneity. These findings indicate advanced thyroid atrophy as a consequence of long-term sunitinib exposure. (**B**) Longitudinal grey-scale ultrasound image of the thyroid gland after three years of sunitinib therapy. The image demonstrates a thin, markedly reduced thyroid lobe with significantly diminished parenchymal volume and decreased echogenicity. The flattened glandular contour reflects advanced thyroid atrophy, consistent with long-term sunitinib-induced structural deterioration.

After three years of ongoing treatment, follow-up imaging confirmed advanced parenchymal loss (total volume: ~2 mL) with profound echogenic reduction and markedly attenuated vascularisation (Figure 5A,B).

Despite pronounced structural deterioration, the patient tolerated sunitinib well, maintained stable oncological status, and continued thyroid hormone replacement with good biochemical control. No further TSH elevations above 10 mIU/L were recorded. Antibody titres remained low and stable.

This case illustrates a characteristic phenotype of sunitinib-induced thyroid dysfunction as follows: early biochemical fluctuations, followed by transition to overt hypothyroidism requiring long-term levothyroxine therapy and accompanied by profound structural thyroid atrophy. The strong chronological relationship between drug exposure, hormonal disturbance, and irreversible gland involution supports a causal mechanism.

## 4. Discussion

Kinases are enzymes responsible for transferring phosphate groups from high-energy compounds such as ATP to protein substrates, modifying protein structure and cellular function [9]. When phosphorylation occurs at tyrosine residues, the enzyme involved is classified as a tyrosine kinase. These molecules play a central role in regulating cell proliferation, differentiation, angiogenesis, apoptosis, and motility, and abnormalities in their activity contribute to oncogenesis [10]. Several tyrosine kinases, including vascular endothelial growth factor receptors (VEGFRs) and platelet-derived growth factor receptors (PDGFRs), function as ligand-activated membrane receptors controlling diverse intracellular signalling pathways [11].

The advent of tyrosine kinase inhibitors (TKIs) has enabled targeted suppression of dysregulated oncogenic signalling. Sunitinib, a multitarget TKI acting on VEGFR and PDGFR, is widely used in renal cell carcinoma and several other malignancies [12]. Although generally well tolerated, increasing clinical evidence indicates that TKI therapy is associated with endocrine disturbances, especially thyroid dysfunction, which is most frequently manifested as hypothyroidism [13].

Importantly, the thyroid gland physiologically expresses multiple receptor tyrosine kinases, including vascular endothelial growth factor receptors (VEGFRs) and platelet-derived growth factor receptors (PDGFRs), which play a key role in maintaining normal thyroid structure and function [13,14]. These receptors regulate thyroid microvascular integrity, angiogenesis, endothelial cell survival, and paracrine signalling within the follicular environment, thereby supporting adequate tissue perfusion and hormonogenic capacity. Consequently, inhibition of tyrosine kinase signalling may directly disrupt thyroid homeostasis, predisposing the gland to ischemic injury, structural involution, and progressive functional decline.

The mechanism underlying sunitinib-associated thyroid dysfunction appears multifactorial. The most widely supported hypothesis involves impaired thyroid vascularisation due to VEGFR and PDGFR inhibition, leading to tissue ischemia, destructive thyroiditis, and irreversible gland atrophy. Imaging studies, including those presented in this report, support the following vascular mechanism: progressive loss of thyroid volume over time strongly suggests parenchymal destruction rather than functional suppression alone [15]. A biphasic biochemical pattern, beginning with fluctuating TSH levels and ultimately progressing to sustained hypothyroidism requiring levothyroxine replacement, has also been described in prior studies and was clearly evident here [16].

Additional contributory pathways have been proposed. These include impaired iodine uptake, inhibition of thyroid peroxidase, altered deiodinase activity affecting peripheral hormone metabolism, and possible disruption of the hypothalamic–pituitary–thyroid axis. Central hypothyroidism, though less common, has also been reported in association with VEGFR inhibition [17]. Similarly, changes in hepatic hormone clearance and gastrointestinal malabsorption may influence levothyroxine dosing requirements, particularly in patients receiving long-term therapy; these effects reflect adaptive alterations in hepatic metabolism and chronic gastrointestinal dysfunction associated with prolonged treatment, resulting in variable hormone bioavailability [18].

From a clinical standpoint, diagnosis can be challenging because the symptoms of thyroid dysfunction overlap with cancer-related fatigue, systemic illness, and treatment toxicity. Consequently, routine thyroid function monitoring before and during TKI therapy is essential. Several guideline statements and observational studies recommend regular biochemical assessment, particularly during early treatment cycles when abnormalities may first emerge [19]. Importantly, hypothyroidism does not generally necessitate modification or interruption of TKI therapy; in most cases, appropriately titrated levothyroxine allows the continuation of treatment without oncological compromise [20].

The course documented in this case—beginning with fluctuating thyroid hormone concentrations, progressing to substantial structural atrophy confirmed by ultrasound and culminating in stable replacement-dependent hypothyroidism—aligns with the evolving understanding that sunitinib-induced thyroid dysfunction reflects a destructive process rather than a transient metabolic disturbance. This reinforces the role of vascular and inflammatory injuries as key drivers of irreversible gland failure in susceptible patients [21].

Recognition of this mechanism is clinically relevant. Proactive monitoring, early initiation of treatment, and continuity of oncologic therapy improve patient safety and reduce the risk of delayed diagnosis. Moreover, hypothyroidism may itself carry prognostic significance: in some malignancies, it has been associated with improved outcomes, raising questions about the interaction between thyroid status and tumour biology [22]. Further research is warranted to clarify whether thyroid dysfunction represents a biomarker of treatment response, a mechanism of toxicity, or both.

## 5. Conclusions

In summary, thyroid dysfunction—particularly hypothyroidism—is a common complication during tyrosine kinase inhibitor therapy, affecting both patients with no prior history of thyroid disease and those without underlying thyroid pathology. Although the pathogenesis is not fully understood, current evidence suggests that vascular injury leading to destructive thyroiditis and progressive gland atrophy, together with altered thyroid hormone metabolism, represents the most plausible mechanism responsible for TKI-associated thyroid dysfunction.

Given the often nonspecific clinical presentation and the potential for progressive and irreversible changes, routine biochemical monitoring is strongly recommended, particularly early in the course of treatment and throughout long-term therapy. When thyroid dysfunction occurs, timely intervention with appropriately titrated levothyroxine enables restoration and maintenance of euthyroidism. Importantly, thyroid abnormalities do not typically necessitate cessation or modification of anticancer therapy, and most patients can continue treatment without compromise to oncologic outcomes.

This case underscores the value of coordinated oncologic and endocrine care, highlights the importance of early recognition, and supports proactive management strategies to minimise symptomatic burden and prevent delayed diagnosis. As TKIs continue to play an expanding role in cancer treatment, further research is needed to clarify long-term endocrine outcomes, identify risk factors for irreversible gland injury, and explore whether TKI-induced hypothyroidism may serve as a clinical or prognostic marker of treatment response.

## Data Availability

All original data supporting the findings of this study are contained within the article. Further inquiries may be directed to the corresponding author.

## Abbreviations

The following abbreviations are used in this manuscript:

ATP	adenosine triphosphate
FT3	free triiodothyronine
FT4	free thyroxine
PDGFR	platelet-derived growth factor receptor
R1	microscopically positive surgical margin
TSH	thyroid-stimulating hormone
TKI	tyrosine kinase inhibitor
VEGFR	vascular endothelial growth factor receptor
